# 

**Published:** 1888-04

**Authors:** 


CONTENTS.

VOL. 35 No. 4.

| | PAGE. |
|---|---|
| Special | 73 |
| The Home | 73 |
| A Neglected Remedy | 75 |
| Suicidal Pin Wounds | 80 |
| Necessary Rules of Sleep | 82 |
| The Bamboo | 83 |
| Heavenly Messengers | 84 |
| How Big are the Waves | 87 |
| Another Georgia Wonder Woman | 88 |
| Influence of Planets | 89 |
| A Doleful Picture | 90 |
| Wonders of Plant Life | 91 |
| Book Notices | 92 |
| Household | 93 |
| Miscellaneous | 95 |

INDEX TO ADVERTISEMENTS OF HALF A PAGE AND OVER.

| | PAGE. |
|---|---|
| Crystal Pepsin Tablets | I |
| Dr. Molesworth | I |
| Stevensville Mills | II |
| The Tea Cure | II |
| The American Oxygen Association | III |
| Warner's Log Cabin Remedies. | IV |
| Lactopeptine | V |
| Right Here in New York | VI |
| Bovinine | VIII |
| $10 Real Value Free | IX |
| Hollow Suppositories | X |
| Aletris Cordial | XI |

